# Anatomical study of jugular foramen in the neck^☆^

**DOI:** 10.1016/j.bjorl.2018.09.004

**Published:** 2018-10-09

**Authors:** Carlos Alberto Ferreira de Freitas, Luiz Roberto Medina dos Santos, Andreza Negreli Santos, Augusto Barreto do Amaral Neto, Lenine Garcia Brandão

**Affiliations:** a Universidade Federal do Mato Grosso do Sul (UFMS), Faculdade de Medicina, Campo Grande, MS, Brazil; b Centro de Pesquisa Oncológica (CEPON), Florianópolis, SC, Brazil; c Universidade de São Paulo (USP), Faculdade de Medicina, São Paulo, SP, Brazil

**Keywords:** Skull base, Jugular veins, Glossopharyngeal nerve, Vagus nerve, Accessory nerve, Base do crânio, Veias jugulares, Nervo glossofaríngeo, Nervo vago, Nervo acessório

## Abstract

**Introduction:**

The anatomical complexity of the jugular foramen makes surgical procedures in this region delicate and difficult. Due to the advances in surgical techniques, approaches to the jugular foramen became more frequent, requiring improvement of the knowledge of this region anatomy.

**Objective:**

To study the anatomy of the jugular foramen, internal jugular vein and glossopharyngeal, vagus and accessory nerves, and to identify the anatomical relationships among these structures in the jugular foramen region and lateral-pharyngeal space.

**Methods:**

A total of 60 sides of 30 non-embalmed cadavers were examined few hours after death. The diameters of the jugular foramen and its anatomical relationships were analyzed.

**Results:**

The diameters of the jugular foramen and internal jugular vein were greater on the right side in most studied specimens. The inferior petrosal sinus ended in the internal jugular vein up to 40 mm below the jugular foramen; in 5% of cases. The glossopharyngeal nerve exhibited an intimate anatomical relationship with the styloglossus muscle after exiting the skull, and the vagal nerve had a similar relationship with the hypoglossal nerve. The accessory nerve passed around the internal jugular vein via its anterior wall in 71.7% of cadavers.

**Conclusion:**

Anatomical variations were found in the dimensions of the jugular foramen and the internal jugular vein, which were larger in size on the right side of most studied bodies; variations also occurred in the trajectory and anatomical relationships of the nerves. The petrosal sinus can join the internal jugular vein below the foramen.

## Resumo

**Introdução:**

A complexidade anatômica do forame jugular torna a realização de procedimentos cirúrgicos nessa região delicada e difícil. Devido aos avanços obtidos nas técnicas cirúrgicas, as abordagens do forame jugular têm sido feitas com maior frequência, o que requer uma melhoria correspondente no conhecimento de sua anatomia.

**Objetivo:**

Estudar a anatomia do forame jugular, da veia jugular interna e dos nervos glossofaríngeo, vago e acessório, assim como as relações anatômicas entre estas estruturas na região do forame jugular e no espaço parafaríngeo.

**Método:**

Foram examinados 60 lados de 30 cadáveres frescos algumas horas após a morte. Os diâmetros e suas relações anatômicas foram analisados.

**Resultados:**

Os diâmetros do forame jugular e da veia jugular interna foram maiores no lado direito na maioria dos espécimes estudados. O seio petroso inferior terminava na veia jugular interna até 40 mm abaixo do forame jugular, em 5% dos casos. O nervo glossofaríngeo exibiu uma relação íntima anatômica com o músculo estiloglosso após a sua saída do crânio e o nervo vago exibiu uma relação semelhante com o nervo hipoglosso. O nervo acessório passou em torno da veia jugular interna via sua parede anterior em 71,7% dos cadáveres.

**Conclusão:**

Foram encontradas variações anatômicas nas dimensões do forame jugular e da veia jugular interna, que apresentaram tamanhos maiores à direita na maioria dos espécimes estudados; variações também ocorreram na trajetória e nas relações anatômicas dos nervos. O seio petroso pode se unir à veia jugular interna abaixo do forame.

## Introduction

The jugular foramen is an opening in the petro-occipital fissure formed by the junction of the petrous portion of the temporal bone and the lateral border of the occipital bone. It is located at the skull base, lateral to the foramen magnum, posterior and medial to the base of the styloid process of the temporal bone, slightly lateral and posterior to the carotid canal and lateral to the hypoglossal nerve canal.^1^, ^2^

The internal jugular vein, the main venous drainage pathway of the brain, and three cranial nerves, the glossopharyngeal nerve or cranial nerve IX, the vagal nerve or cranial nerve X and the accessory or cranial nerve XI, pass through the jugular foramen, also called the posterior lace rum,^3^ to reach the neck. The inferior petrosal sinus, which drains blood from the cavernous sinus to the jugular bulb or to the internal jugular vein itself, and one or more branches of the ascending pharyngeal artery or the occipital artery to the meninges,^1^, ^2^, ^4^ are also found in the jugular foramen region.

This anatomical complexity makes performing surgical procedures in this region delicate and difficult.^5^ The anatomical relationships of the structures emerging from the lower opening of the jugular foramen and their relationships in the lateral-pharyngeal space are of great interest in the fields of head and neck and skull base surgery. This study was performed with the objective of studying the anatomy of the jugular foramen, the internal jugular vein and the glossopharyngeal, vagal and accessory nerves in the jugular foramen region and lateral-pharyngeal space.

## Methods

A total of 60 dissections were performed on 30 non-embalmed adult cadavers, prior to autopsy. This study was approved by the institution's research committee and is in accordance with the ethical and methodological standards.

The group consisted of 36 (60%) dissections of 18 male corpses and 24 (40%) dissections of 12 female corpses. The age distribution ranged from 34 to 87 years, with a median of 58 years, mean of 60 years and standard deviation of 17.34 years. With regard to the studied side, 30 (50%) were left side dissections, and 30 (50%) were right side dissections. Regarding skin color, 28 (46.7%) dissections were performed on white cadavers, and 32 (53.3%) were performed on non-white cadavers. The physical type was also observed, with 16 longilineal (26.7%) and 44 normolineal (73.3%) bodies. None of the bodies were classified as brevilineal.

The study included only cadavers without diseases of the neck that could interfere with the anatomy of the analyzed structures, such as those with tumors, those in which death had been due to traumatic causes and those with gross deformities of the cervical region. The study was performed on non-embalmed cadavers a few hours after death so that the results would reflect realistic conditions as closely as possible. After careful dissection of the area and exposure of the studied structures, the dimensions, trajectory and anatomical relationships were noted.

## Results

**Figure 1:**
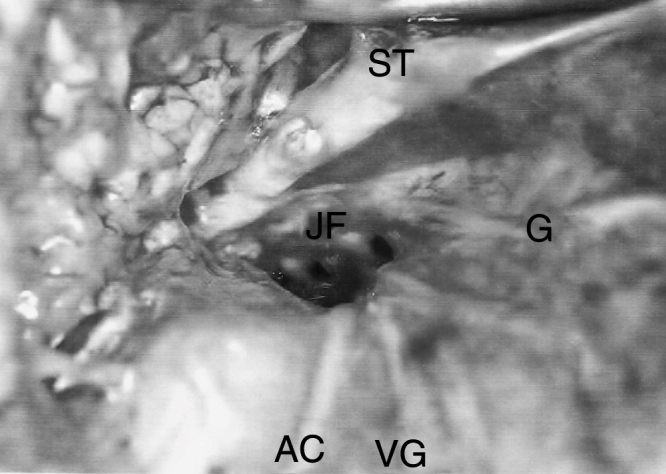
Right jugular foramen (JF) after internal jugular vein remotion, accessory nerve (AC), glossopharyngeal nerve (G), vagus nerve (VG), styloid process (ST).

The lower opening of the jugular foramen had the largest diameter, ranging from 2.8 mm to 13 mm, with a median of 9.0 mm, mean of 8.5 mm and standard deviation of 2.53 mm (Fig. 1). In 73.3% of the studied cadavers, the right side was larger than the left.

**Figure 2:**
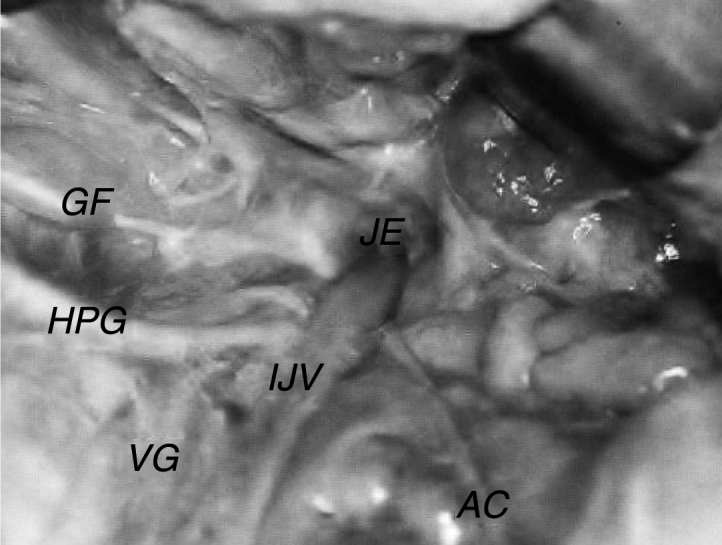
Left accessory nerve (AC) crossing posteriorly to the internal jugular vein (IJV). JF, jugular foramen; G, glossopharyngeal nerve; HPG, hypoglossal nerve; VG, vagus nerve.

The internal jugular vein exhibited diameters of 2.5–9.0 mm with a median of 6.0 mm, mean of 6.2 mm and standard deviation of 1.81 mm. In 66.6% of the specimens, the vein was larger on the right side, 10% had equal diameters, and in 23.4%, the internal jugular vein was larger on the left side (Fig. 2).

**Figure 3:**
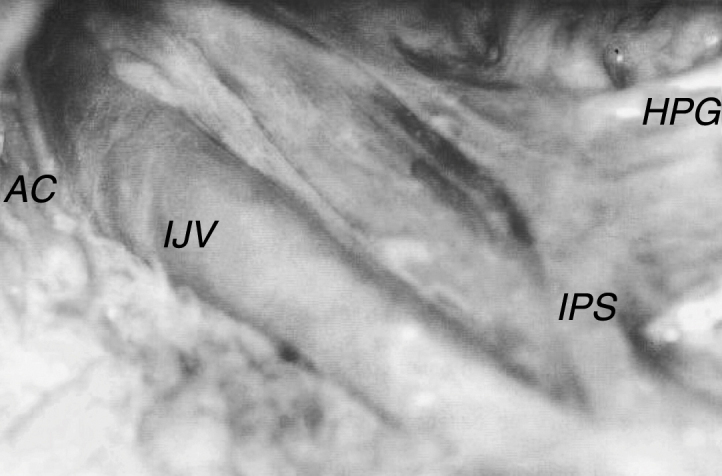
Right inferior petrosal sinus (IPS) in the neck, accessory nerve (AC) crossing in front of the internal jugular vein (IJV). HPG, hypoglossal nerve.

The inferior petrosal sinus was found below the lower opening of the jugular foramen in 5% of the studied specimens (3 cases from a total of 60). It was located on the right side, occupying the anterior and medial portion of the foramen between the glossopharyngeal and vagal nerves (Fig. 3) and ending in a distance varying between 23 and 40 mm below the foramen, with a diameter of between 2.0 and 4.0 mm.

The glossopharyngeal nerve exhibited diameters of 0.6–2.0 mm, with a median of 1.0 mm, mean of 1.1 mm and standard deviation of 0.3 mm. The nerve emerged from the jugular foramen in all cases studied via the anterior and medial portion, in front of the vagal nerve, and curved medially, near the posterior and inferior face of the styloglossus muscle and the anterior wall of the internal carotid artery (Fig. 2).

The vagus nerve exhibited diameters of 1.2–3.8 mm, with a median of 2.4 mm, mean of 2.4 mm and standard deviation of 0.45 mm. In all cases studied, the nerve emerged from the jugular foramen via the anterior and medial portion, between the glossopharyngeal and accessory nerves. In all specimens, the vagus nerve exhibited a close relationship with the hypoglossal nerve, which, after exiting the skull, continued around the vagus nerve via its inferior ganglion, between the internal jugular vein and internal carotid artery, following its trajectory toward the base of the tongue (Fig. 2).

The accessory nerve exhibited diameters of 0.9–2.0 mm, with a median of 1.2 mm, mean of 1.3 mm and standard deviation of 0.34 mm. In all cases studied, the nerve emerged from the jugular foramen via the anterior and medial portion, posterior to the vagal nerve. After exiting the skull, it followed a different trajectory in relation to the internal jugular vein. In 71.7% of cases, the accessory nerve passed around the anterior wall (Fig. 3), and in 28.3% of cases, it passed around the posterior wall (Fig. 2).

## Discussion

The diameter of the jugular foramen was greater on the right side in 73.3% of the cadavers analyzed in this study. Others authors have found similar results.^2^, ^4^, ^5^, ^6^, ^7^, ^8^, ^9^

The most important conclusion of this study was the finding of a Petrosal Lower Sinus in the neck. This malformation can be an important cause of bleeding during surgical procedures in the region.

Extracranial extension of the inferior petrosal sinus ending in the internal jugular vein below the jugular foramen is uncommon. Such a situation was observed by Gailloud et al.^10^ in 5 of 101 patients studied. According to these authors, this structure should be called the accessory jugular vein. For Miller et al.,^11^ in 10% of cases studied, the inferior petrosal sinus joined the internal jugular vein via a small vein in the region corresponding to the middle portion of the atlas transverse process.

In turn, Katsuta et al.^4^ noted that the lower petrosal sinus drains blood into the jugular bulb through multiple channels; the largest has a diameter of between 2.0 and 3.0 mm and passes between the glossopharyngeal nerve, anteriorly, and the vagal nerve, posteriorly. They also stated that, rarely, this main sinus channel drains into the internal jugular vein below the extra cranial opening of the jugular foramen.

Rubinstein et al.^12^ confirmed that, as reported in other studies, the area where the inferior petrosal sinus drains the blood into the jugular venous system is variable and can occur within the foramen, in the lower opening, or below the opening.

Lv and Wu^13^ described a duplication of the upper part of the internal jugular vein, separated by the accessory nerve, in 0.4% of cases. Mitsuhashi^14^ found the end of the inferior petrosal sinus below the jugular foramen in 37.3% of the studied cases. Zhang et al.^15^ found this change in 34.6% of cases, with a mean diameter of 2.51 mm and ending 40 mm from the jugular foramen.

In our sample, this anatomical change was found in 5% of cases, all on the right side, with a cervical extension of 23.0–40.0 mm and a diameter ranging between 2.0 and 4.0 mm. The vein exited the skull via the region medial and anterior to the jugular foramen, posterior to the glossopharyngeal nerve and in front of the vagus nerve, confirming the findings of other studies.

Call and Pullec,^16^ Petriglieri^17^ and Rhoton and Buza^7^ reported an anatomical variation in which the glossopharyngeal nerve exits the skull via a channel of its own. This variation was not found in any of the cadavers we studied. Tubbs et al.^18^ found a meningeal septum separating the glossopharyngeal nerve from the vagal and accessory nerves in 36% of cases, and of these, 7.2% were ossified. We found a mean glossopharyngeal nerve diameter of 1.1 mm. Tekdemir et al.^19^ found a mean diameter of 2.3 mm.

After exiting the skull, the accessory nerve exhibits a variable trajectory in relation to the internal jugular vein. This nerve curves laterally at a level corresponding to the transverse apophysis of the atlas and crossing the internal jugular vein most often via its front wall. We found this relationship in 71.7% of the cases studied. In the remaining 28.3% of cases, the nerve passed behind the vein. Katsuta et al.^4^ and Cock^20^ have found similar results.

Parsons and Keith, cited by Cock,^20^ obtained a similar result, although they observed a modification in which, in 3.2% of the 415 skull sides studied, cranial nerve XI passed through the jugular vein. Hinsley^21^ also found one case out of 116 in which the accessory nerve passed through the jugular vein at the height of the posterior belly of the digastrics muscle. We found no such variation in our sample.

Caliot et al.^22^ found a higher frequency (90%) of cases with the nerve in front of the vein. They also reported that the accessory nerve was posterior to the internal jugular vein on both sides in only one case. Some other studies, however, have found a slight predominance of the accessory nerve crossing the vein laterally via its anterior wall. Diop et al.^23^ found a rate of 57.6%, and Saleh et al.^24^ 52.5%. This occurs at a level corresponding to the atlas transverse process, as we also confirmed in our study.

A similar result was obtained by Soo et al.,^25^ with 56% and 44% of nerves passing anteriorly and posteriorly, respectively. Berrone et al.^26^ also found an accessory nerve with a “pre-venous” trajectory in slightly more than 50% of cases and a “retro-venous” trajectory in the remaining cases.

Lee et al.^27^ studied the relationship between the accessory nerve and the internal jugular vein in 181 patients undergoing neck dissection and found a slightly different result, with the nerve in a position dorsal to the internal jugular vein in 57.4% of cases and with the nerve passing through the internal jugular vein in 2.8% of cases. Piffer et al.^28^ analyzed 32 cadaver sides and also reported finding the accessory nerve in a dorsal position in 81.2% of cases.

## Conclusion

The results of this study showed that important anatomical variations exist in the lower opening of the jugular foramen. The diameters of the jugular foramen and internal jugular vein were variable and were greater on the right side in most of the studied specimens. The inferior petrosal sinus may end in the internal jugular vein up to 40 mm below the jugular foramen in up to 5% of cases. The vagus nerve is closely related to the hypoglossal nerve, with the latter having its own channel for exiting the skull. The glossopharyngeal nerve has an intimate anatomical relationship with the styloglossus muscle after it exits the skull. The accessory nerve more frequently passed around the internal jugular vein via its anterior wall and less often via its posterior wall.

## Conflicts of interest

The authors declare no conflicts of interest.
